# AI and Machine Learning Terminology in Medicine, Psychology, and Social Sciences: Tutorial and Practical Recommendations

**DOI:** 10.2196/66100

**Published:** 2025-08-18

**Authors:** Bo Cao, Russell Greiner, Andrew Greenshaw, Jie Sui

**Affiliations:** 1 Department of Psychiatry, University of Alberta, 4-142A Katz Group Centre for Research, 11315 - 87 Ave NW, Edmonton, AB, T6G 2B7, Canada, 1 7804929576; 2 Department of Computing Science, Faculty of Science, University of Alberta, Edmonton, AB, Canada; 3 School of Public Health, University of Alberta, Edmonton, AB, Canada; 4 Alberta Machine Intelligence Institute (Amii), Edmonton, AB, Canada; 5 School of Psychology, University of Aberdeen, Aberdeen, United Kingdom

**Keywords:** artificial intelligence, machine learning, terminology, medicine, psychology, social sciences, prediction, regression, deep learning, tutorial, prospective prediction, validation

## Abstract

Recent applications of artificial intelligence (AI) and machine learning in medicine, psychology, and social sciences have led to common terminological confusions. In this paper, we review emerging evidence from systematic reviews documenting widespread misuse of key terms, particularly “prediction” being applied to studies merely demonstrating association or retrospective analysis. We clarify when “prediction” should be used and recommend using “prospective prediction” for future prediction; explain validation procedures essential for model generalizability; discuss overfitting and generalization in machine learning and traditional regression methods; clarify relationships between features, independent variables, predictors, risk factors, and causal factors; and clarify the hierarchical relationship between AI, machine learning, deep learning, large language models, and generative AI. We provide evidence-based recommendations for terminology use that can facilitate clearer communication among researchers from different disciplines and between the research community and the public, ultimately advancing the rigorous application of AI in medicine, psychology, and social sciences.

## Challenges

The rapid growth of artificial intelligence (AI) and machine learning (ML) in medicine, psychology, and social sciences has led to a proliferation of terminology that is often inconsistently and imprecisely applied [1,2]. This inconsistency creates confusion for researchers, clinicians, and policy makers who need to interpret and apply these technologies. Perhaps the most notable example of terminological confusion in the field involves the concept of “prediction,” which is frequently misused in the literature [3,4]. However, similar confusion extends to other key terms such as “validation,” “features,” and even the distinctions between AI, ML, and deep learning (DL) [1].

The confusion about “prediction” is particularly widespread and merits special attention. Yarkoni and Westfall [5] have argued that psychology’s emphasis on explaining the causes of behavior rather than predicting future behavior has led to research programs that provide intricate theories but have little ability to predict future behaviors with appreciable accuracy. McCall et al [4] highlight this issue in the context of sports science and medicine, noting that much of the confusion stems from a mismatch between statistical modeling and subsequent interpretation of findings. After examining the literature in sports science that claimed to predict performance, talent, or injury, they found that the vast majority of studies actually analyzed association rather than prediction. This confusion can have serious consequences for practitioners who rely on these studies to make decisions about athlete training, selection, or rehabilitation. Varga et al [3] conducted a systematic review in the field of diabetes and found that the term “prediction” is often incorrectly used to refer to association statistics. The confusion between association and prediction can lead to inflated expectations about the capabilities of models, and even biomarkers with strong statistical associations, causal biomarkers, or biomarkers with clear roles in disease pathophysiology can be poor predictors of future disease in individuals [3].

In addition to misinterpreting association as predictions, the term “prediction” has been frequently misused as a marketing hot term for retrospective observational studies that lack external or future (prospective) validation in broader medical literature. In a review of 152 ML studies, Navarro et al [2] found that most ML prediction model studies suffered from poor methodology and reporting. They noted that the majority of these studies were retrospective in nature and lacked proper external validation (87% with only internal validation). Ramspek et al [6] noted that external validation remains rare in prediction model studies, with few models being validated in independent datasets. This lack of validation raises serious concerns about the generalizability of these models to new populations or settings and true predictions for future outcomes. Similarly, Nagendran et al [7] found that of 81 nonrandomized clinical trials identified using DL, only 9 were prospective and 6 were tested in a real-world clinical settin, and Abdulazeem et al [8] found only 8 out of 106 (7.6%) ML studies in primary health care were prospective in desgin.

According to all the aforementioned studies, there have been at least four distinct categories of studies that are commonly conflated under the umbrella of “prediction.” (1) Association studies (correlation or risk factor analysis mislabeled as “prediction”). (2) Retrospective modeling without external validation (models aiming to “predict” unknown outcomes and developed on an existing dataset without external or future validation), including studies showing prediction metrics without mentioning validation. (3) Retrospective modeling with external validation (models aiming to “predict” unknown outcomes and developed on existing data with external validation on an independent dataset but without future prediction). (4) Prospective prediction studies (models predicting future outcomes on new data or through a prospective design).

Table 1 summarizes the prevalence of these categories across domains based on systematic reviews and perspective papers from a sample of papers from medicine and psychology literature reporting potential issues of “prediction” studies. This table reveals several concerning trends.

**Table 1.** Prevalence of different types of studies labeled as “prediction.”

Domain	Association studies	Retrospective without external validation	Retrospective with external validation	Prospective prediction	Reference
Review and perspective studies on prediction					
Diabetes	61%	39%	N/A^a^	N/A	Varga et al [3]
Psychology	Widespread	Widespread	Rare	N/A	Yarkoni and Westfall [5]
Sports science	77% (performance), 90% (talent), and 65% (injury)	10%‐35%	N/A	N/A	McCall et al [4]
Review studies on machine learning					
Clinical prediction	N/A	Most	0.5% in 1990 7% by 2019	N/A	Ramspek et al [6]
Health care	N/A	87.00%	13.00%	N/A	Navarro et al [2]
Medical imaging	N/A	45.70%	43.00%	11.00%	Nagendran et al [7]
Primary health care	N/A	76.50%	24%	7.60%	Abdulazeem et al [8]

a Not applicable.

First, the majority of studies labeled as “prediction” are actually association studies, characterized by the absence of model validation with new or unseen data. Second, these studies discuss the confusion surrounding other key terminological confusions that are relevant to but beyond “prediction”; for example, there are often misunderstandings of validation techniques, the assessment of generalizability [2,5], and the role of features, and independent variables. Finally, there is considerable confusion about the distinctions between traditional statistical approaches such as linear regression and contemporary ML techniques. It is important to note that such categories may not reflect the crucial temporal essence of prediction (eg, predicting future outcomes) or the predictability or generalizability of a model (eg, the ability to predict the outcome on validation data collected in the future). Additionally, there is also confusion about the basic terminology of AI methodologies. Terms such as “artificial intelligence,” “machine learning,” and “deep learning” are often used without appropriate definitional boundaries or used interchangeably. This terminological confusion has significant implications for research, practice, and policy in medicine, psychology, and social sciences. It can lead to misleading claims about model performance, inappropriate application of models in clinical settings, and inefficient use of research resources. As AI and ML continue to permeate these fields, there is an urgent need for greater clarity and consistency in the use of terminology.

In summary, most “prediction” studies predominantly explain the relationships between variables and outcomes within existing data, rather than establishing generalizable prediction through rigorous validation with new data. Even among studies using appropriate ML prediction models with both training and validation components, most implement only basic predictive methodologies, focusing on prediction with retrospective modeling and lacking external validation. True prospective prediction studies remain rare, with comprehensive explanations of prediction terminologies absent (see section “Prediction”).

The following sections will examine these terminological challenges and provide practical recommendations. We begin with the concept of prediction, with a focus on the temporal perspective of prospective prediction and offering operational implementation tutorial (see section “Prediction”), followed by testing and validation methodologies in the context of evaluating the generalizability of prediction (see section “Testing, Validation, Cross-Validation, and External Validation”), addressing overfitting phenomena, and comparing regular and ML regression models (see section “Overfitting, Linear Regression, Regularization, and ML”), distinctions between features, independent variables, predictors, risk factors, and causal factors (see section “Feature, Independent Variable, Predictor, Risk Factor, and Causal Factor”), and finally, clarification of different AI methodologies (see section “AI, ML, DL, Large Language Models, and Generative AI”). Through this terminological precision, we aim to provide a foundation for more rigorous and transparent interdisciplinary research that offers practical implementation pathways for AI applications in medicine, psychology, and social sciences.

## Prediction

### Overview

The term “prediction” has been widely used in different disciplines, while no real prediction of future outcomes is present [3,5]. Historically, a regression or correlation model may be considered in medicine, psychology, and social sciences as a prediction model [9] simply because it reflects the fit or mathematical modeling between independent variables (eg, X, a set of clinical or cognitive assessments, or brain structure or activity) and dependent variables (eg, Y, the risk for a specific mental disorder, or social behavior or norms). This is reasonable, as the model can inform us about the potential value of Y when we have an observation of X. However, such models and analyses usually cannot be generalized to individual-level prediction on new data [3,5,10] (see section “Overfitting, Linear Regression, Regularization, and ML”).

Unlike traditional regression and correlation methods that primarily focus on identifying relationships between variables and outcomes within a single dataset, ML algorithms—both classification (predicting binary or categorical outcomes) and regression (predicting continuous outcomes; see section “Overfitting, Linear Regression, Regularization, and ML”)—are designed explicitly with generalization in mind. They typically involve validation procedures (eg, training models on a subset of data and validating its performance on an unseen subset; see section “Testing, Validation, Cross-Validation, and External Validation”), enabling robust prediction of new or future outcomes across individuals or within individuals over time [11]. In this paper, we will focus on the prediction of an explicitly measurable outcome that can be used to verify the prediction (eg, a diagnosis or treatment outcome), as commonly considered as supervised learning in ML (compared with unsupervised learning, eg, clustering) [12].

With increasing adoption of ML in research and practice in medicine, psychology, and social sciences, using the term “prediction” for the fit of a regression or correlation model is often no longer appropriate, and prediction, as its literal meaning, should have a component that is new to the existing observations or assessments. A summarized fitting of a mathematical model between variables in an existing dataset should be referred to as a fitting or explanatory model, rather than a prediction model, until it is validated on new data, and the corresponding study should be referred to as an association study.

**Figure 1.**
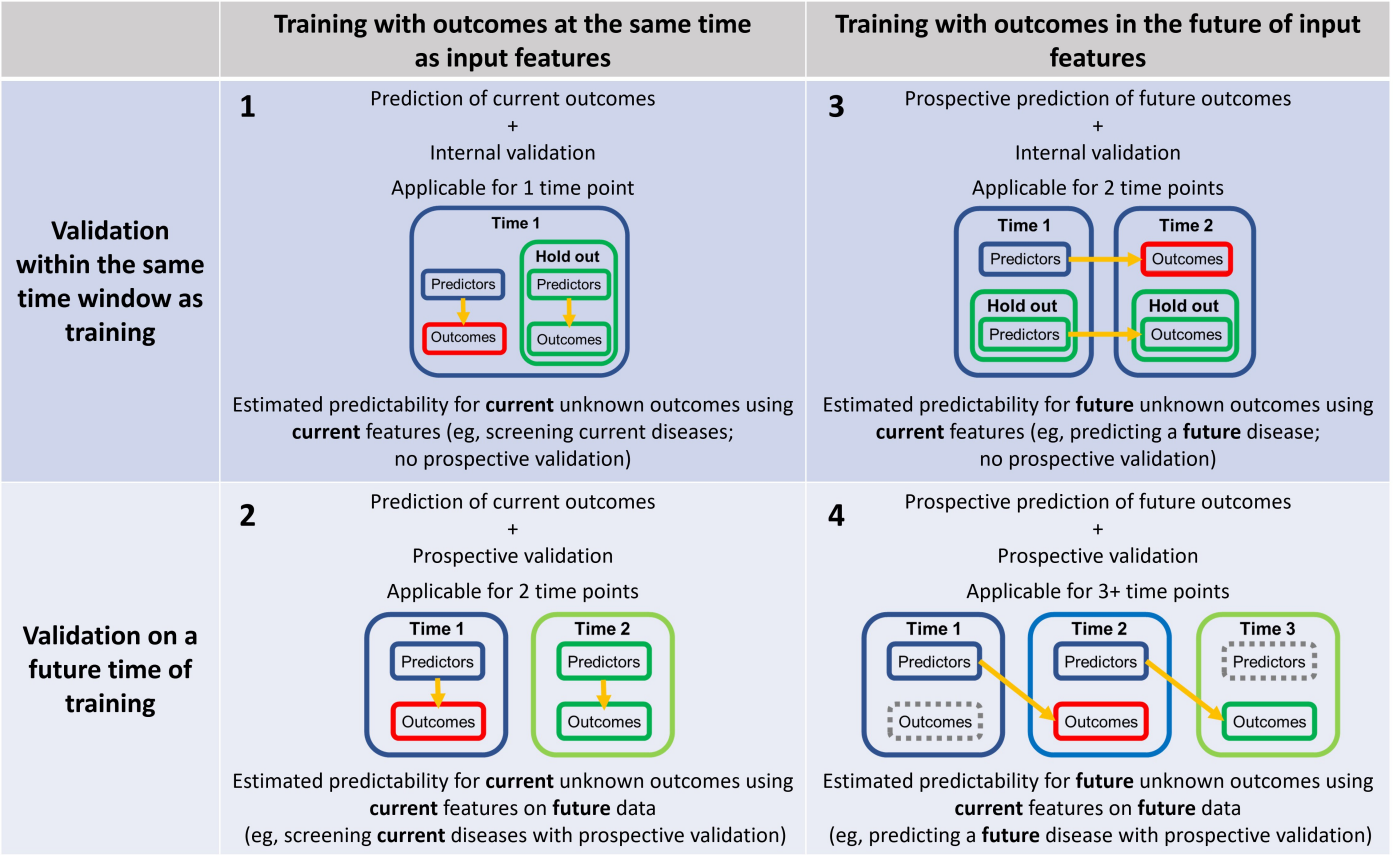
Prediction of current and future outcomes with current and future validation.

In practice, we should refer to an outcome generated by a model as a “prediction” only when the outcome has not been observed yet and can only be obtained in the future. Ideally, this should be the case. However, future observations with sufficient validity may be challenging and take time to obtain and, often, not feasible for secondary data analysis on existing data from a research team that is not the original data owner. Given these practical constraints, many models and validation techniques can provide reasonable estimation about predictions to a different population (eg, models developed with a younger adult dataset generalized to older populations [13]) or the same sample over time [14], and we recommend that “prediction” should include scenarios when we can show evidence of how a model will perform on new data relative to the data used to develop the model. The new data need not be strictly “future” compared with the existing data but must remain previously unseen by the model during model training. With this expanded operational definition, we can use the term “prediction” for outcomes generated by models trained with existing data and then applied to new data, while we recommend using *Prospective Prediction (or Forecasting*) to specifically refer to the predictions aiming for future outcomes to emphasize the temporal aspect of the prediction [14]. The “future” can be relative to the time of model training or the time of data collection of predicting variables in a retrospective dataset, where the validation data are held out or collected already at a later time, or relative to the current time, when the validation data are not yet collected. Thus, it is important for researchers to explicitly report the specific characteristics of the “prediction” as summarized in Figure 1, which distinguishes 4 types of prediction based on temporal relationships between training and validation. We focus on internal validation here, but external validation can be applied to replace the holdout internal validation data or be used as fully independent validation data (Table S1 in Multimedia Appendix 1).

1. The ML model itself, with internal validation, is trained only on current outcomes observed within the same time frame of the variables used to predict the unknown outcomes (input to the ML model, ie, features; see section “Feature, Independent Variable, Predictor, Risk Factor, and Causal Factor”). Its predictability is estimated through the validation of the predicted outcomes using current features on a holdout dataset from the same dataset within the same time frame. Such a model and procedure can be used to develop prediction tools (eg, screening) for a current disease using assessments performed at the same time frame, but it needs further longitudinal validation on future data to confirm its generalizability over time or further external validation to confirm its generalizability across populations (Table S1 in Multimedia Appendix 1).
2. The ML model itself is trained only on current outcomes observed within the same time window of features, and its predictability is estimated through the validation of the predicted outcomes using features on a future collection of data (eg, time 2). Such a future collection can be from the same people (eg, a whole population) or an independent cohort. Such a model and procedure can be used to develop prediction tools for a current disease using assessments collected at the same time frame, and its predictability is validated by future data, which greatly strengthens its potential application. However, such a model is not designed to predict future outcomes as their training design remains anchored to the relationships between variables and outcomes collected in the same time frame.
3. The ML model is trained on outcomes observed in a later time frame of the features. Its predictability is estimated through the validation of the predicted future outcomes (eg, time 2) using current predictors (eg, time 1) on a holdout dataset from the same dataset. Such a model and procedure can be used to develop prediction tools for a future disease (eg, onset of major depressive or anxiety disorder [15,16]) using assessments collected at an earlier time point. While providing valuable predictive potential, further longitudinal validation of future data is needed.
4. The ML model is trained on outcomes observed in a later time frame of the features, and its predictability is estimated through the validation of the predicted outcomes using features collected in future (eg, time 2) and outcomes observed in an even later time frame of the features (eg, time 3). Such a model and procedure can be used to develop prediction tools for a future disease or disease progression using earlier assessments, and its predictability is validated by future data, which greatly strengthens its potential application for real-world forecasting. This approach can also be applied to data collections that do not have discrete time points (eg, streaming data [17]), as long as there are clear time stamps for features and outcomes.

One particular strength of ML approaches over traditional statistical analyses is that they provide predictions at the individual level rather than merely establishing group-level statistical significance. For example, in a typical statistical *t* test, the difference between the means of 2 distributions of an assessment from 2 populations (eg, patients and healthy subjects) is tested against the null hypothesis that the means are not different. Typically, as we have more observations, we will be more confident in estimating the means, thus reaching a higher significance level. However, this increased significance usually does not provide us with a better way to identify a new individual case randomly picked from the 2 populations (eg, patients vs healthy subjects) based on this assessment, because such identification depends only on the discrimination capability between the distributions of the original assessment metrics between the 2 populations and not the distributions of their means. Thus, a higher significance level of a variable at the group level does not automatically lead to better distinction of individual cases between 2 groups or make the variable a better predictor for one of the groups [18]. On the other hand, ML can identify individual cases rather than group characteristic differences [19-23]. In many practical scenarios, identifying individual cases based on high-dimensional potential predicting variables is a much more difficult task than finding significant differences in these variables at the group level [18]. However, finding statistically significant differences in variables could unveil the mechanisms associated with an outcome (eg, disease) and could be a complementary preliminary step for further individualized prediction and risk factor identification. Therefore, we recommend using the term Individualized Prediction for predictions at the individual level in general, as opposed to predictions of outcomes at the group level or population level.

Key terms related to “Individualized Prediction” include “Personalized Medicine” and “Precision Medicine,” which refer to treatment optimizations that consider all personal information. Personalized medicine and precision medicine usually need to engage multiple individualized prediction models for treatment outcome predictions of multiple or all candidate treatment options using multimodal data [24] and thus should not be used casually if the model provides only 1 individual-level prediction for 1 condition or treatment. Currently, precision medicine is sometimes used to refer to the identification of individuals fitting to a highly specific treatment that can be further personalized (eg, gene or stem cell therapy); however, in general, this process usually involves previous attempts of or implicit comparisons with other treatment options.

### Recommendations for the Use of “Prediction”

**Figure 2.**
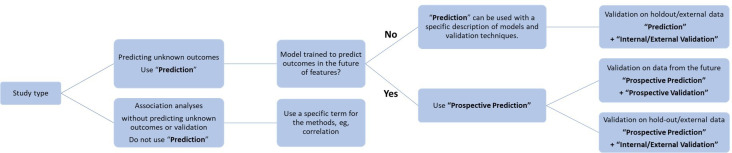
Recommended decision process for the term “prediction” and relevant validations.

The use of the term “Prediction” should consider the following recommendations. First, do not use the term “prediction” for an association study that does not predict any unknown outcomes (eg, correlation) or has no validation procedure to estimate the predictability of the estimated unknown outcome (eg, regression without proper validations). Second, if the model used in the study is trained to predict outcomes in the future of the input features, use the term “Prospective Prediction.*” *“Prediction” can be used if the model developed in the study is trained to predict unknown outcomes in the same time frame as the input features, with proper validation (Figure 2). Specify whether the validation is from a future collection (eg, “prospective validation”), a holdout of the same dataset (eg, “internal validation” and be specific about the validation methods), or an independent dataset (eg, “external validation”). Specify whether the “future” collection is a true prospective design, where the validation dataset is not yet collected, or is from retrospective data that were collected in the future of model training. External and prospective validation will greatly strengthen confidence in the generalizability and predictability of the model’s performance. Third, the term “Individualized Prediction” can be used to differentiate predictions at the individual level from those focusing on outcomes at the group or population level. However, “Personalized Medicine” and ’Precision Medicine” should not be used casually if the model provides only 1 individual-level prediction for a single condition or treatment.

## Testing, Validation, Cross-Validation, and External Validation

### Overview

The ability of ML models to predict outcomes in new data must be demonstrated through a validation process. However, there is common confusion about the rationale for and implementation of testing and validation and the difference between the 2 terms. In ML, it is typical to test the model on a separate dataset. We usually train (or develop) the ML model on a dataset that we call the training set and then test it on another dataset that we call the testing set. Usually, the testing set is an independent data collection similar to the training set. In the case of 1 dataset, we can still try to mimic the training and testing procedure by separating the data into the training data and validation data, with the former used for training the model and the latter for internal testing of the trained model. Sometimes, we can perform the validation in a way that we randomly separate the data into certain portions and use 1 portion as the validation data each time until we exhaust the data, a common procedure that we refer to as cross-validation [25]. Cross-validation is also used for model and variable selection (or, in more common terms, “feature selection”; see section “Feature, Independent Variable, Predictor, Risk Factor, and Causal Factor” about “feature”) [26]. The most important aspect of validation and testing is that the model being trained should not encounter any of the validation data per se (comprising the validation) or any information specifically derived from validation data, including feature selection (eg, potential predicting variables), hyperparameter tuning, and model fitting or optimization. Testing and validation are sometimes used interchangeably. However, in the current literature of medicine, psychology, and social sciences, where prospective studies are still scarce while retrospective studies are dominant, it is usually more appropriate to report the specific validation procedures rather than use the term “testing” generally.

Note that the procedure of testing and validation is important to estimate how the model would perform on new, unseen data, but it is not and should not be a procedure exclusive to ML. For any scientific analysis, we want “predictive results” to be generalizable beyond our own study samples. The procedure of validation, including cross-validation, may not be perfect, especially when implemented improperly, as overestimation and bias still occur depending on how representative the testing and validation data are, but it is a crucial gateway for further real-world implementation of any model [26].

### Recommendations for the Use of “Validation”

It is important to explicitly report whether models have undergone proper validation on independent datasets or cross-validation or holdout validation methods that strictly separate training and validation data. We recommend using specific terms about how validation is performed instead of using the term “testing” or “validation” in general. For example, if the model is developed on 1 dataset and validated on the same data using cross-validation, the term “testing” should be avoided, and the specific cross-validation should be clearly mentioned, for example, 5-fold cross-validation or leave-one-out cross-validation. If the model is developed on 1 dataset and validated on another independent dataset, usually collected from a separate cohort or another geographical region or facility, the term “External Validation” should be used. If the model is developed on 1 population dataset and validated in the future, where prospective prediction and longitudinal collection of the same cohort are usually involved, the term “Prospective Validation” should be used ( Figure 1 and Table S1 in Multimedia Appendix 1). Note that if the validation is performed on independent data collected in the future of training data, the validation is both prospective and external. In all the validation scenarios, the term “testing” is less relevant in medicine, psychology, and social sciences, especially for retrospective studies, compared to in computer science, and specific “validation” is always preferred.

## Overfitting, Linear Regression, Regularization, and ML

### Overview

Validating ML models on new data is essential to demonstrate their generalizability beyond the training data. Without validation, models risk overfitting [10]—a common problem where algorithms learn to fit both the meaningful patterns (“signal”) and random variations (“noise”) in the training data, limiting their applicability to new data. This issue is particularly relevant for linear regression models commonly used in medicine, psychology, and social sciences [27], especially when the number of features is large while the number of observations is limited. Regularized linear regression models (eg, LASSO [Least Absolute Shrinkage and Selection Operator] and elastic net [28]) address this challenge by controlling coefficient magnitudes to prevent overfitting, a principle that extends to other ML approaches [29].

When we compare regularized linear regression algorithms with traditional linear regression in medical, psychological, and social applications, their primary advantage lies in their enhanced generalizability. These ML techniques use regularization to constrain coefficients and optimize them in ways that reduce overfitting [30]. Unlike traditional linear regression, regularization parameters in the regularized linear regression can be automatically learned from training data (and thus considered as ML). While traditional approaches can implement similar constraints through techniques such as the Bayesian information criterion and Akaike’s information criterion [31], regularized methods typically offer superior generalization, although exceptions exist and should be verified through proper validation [32]. The distinction between traditional statistics and ML continues to blur as classical regression models increasingly incorporate regularization techniques, yet meaningful differences persist [33].

Beyond linear approaches, ML offers powerful techniques for capturing nonlinear and complex relationships between features. Methods such as support vector machines with nonlinear kernels [34], random forests [35], and neural networks [29,36] can model intricate patterns and interactions that linear models cannot accommodate. These algorithms automatically discover complex feature combinations and transformations, enabling them to represent relationships that would otherwise require extensive manual feature engineering when using traditional statistical approaches [37,38].

### Recommendations for Reporting Prediction Performance and Model Choices to Prevent Overfitting

With proper validation, overfitting can be recognized during the validation stage. For example, the model may achieve a high accuracy of prediction on the training data but almost a random performance (eg, 50% in a binary classification task) on the validation data. Thus, it is important to report appropriate prediction performance metrics on both the training and especially validation data, including at least sensitivity, specificity, accuracy, precision, and the area under the receiver operating characteristic curve [39]. When possible, external validation is always preferred.

Maintaining transparency about model choices during training and validation is crucial. Linear models are often preferred for their simplicity and explainability in clinical applications [40,41]. However, when more complex processes such as model selection, parameter tuning, and feature engineering are used, researchers should clearly document and justify these steps using flowcharts or concise textual descriptions [42,43]. This kind of rationale is increasingly neglected, partly due to unrealistic expectations that DL models can produce impressive results simply by feeding in complex data. Nonetheless, it is critical to justify every decision in model development—even those involving seemingly opaque parameters—because added complexity can compromise both reproducibility and explainability. Importantly, all selection processes must be performed exclusively within the training stage, using nested cross-validation approaches when necessary, to prevent overfitting and information leakage that could artificially inflate performance metrics [26,44].

## Feature, Independent Variable, Predictor, Risk Factor, and Causal Factor

### Overview

Terminology confusion persists regarding concepts, such as features, independent variables, predictors, risk factors, and causal factors. The term “feature” predominates in ML literature, while “independent variable” is more common in traditional statistics [37,45]. Independent variables are designed to be independent of other variables and serve to explain or predict the dependent variables in statistical models [46]. When these variables are established as influencing an outcome, they may be termed “predictors.”

ML experts typically use “feature” to refer to all input variables in a dataset (excluding the target variable being predicted), with the collection of all possible feature values constituting the “feature space” [47]. In ML prediction models, features function as potential predictors for the target outcome variable, analogous to how independent variables relate to dependent variables in statistical modeling [48], although the features are not necessarily independent of each other.

### Recommendations for the Use of “Feature,” “Independent Variable,” “Predictor,” “Risk Factor,” and “Causal Factor”

**Figure 3.**
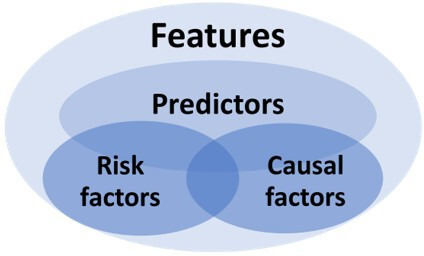
Relationships between features, predictors, risk factors, and causal factors.

We recommend using “Feature” specifically for input variables in ML models, “Independent Variables” in statistical modeling contexts, and reserving “Predictor” for features or independent variables confirmed through rigorous validation to have predictive value for the outcome [49]. Importantly, predictors should not be conflated with risk factors (or protective factors; referred to as risk factors in general) or causal factors [3]. Risk factors typically emerge from association studies or require additional post hoc analysis to confirm their contribution to outcomes [50]. Causal factors, by contrast, demand controlled experimental designs that can establish causative relationships [51]. In general, features include all potential predictors, risk factors, and causal factors, and some predictors could be potential risk factors and causal factors and vice versa (Figure 3). Risk factors or causal factors are not naturally predictors and, similarly, predictors are not always risk factors or causal factors, especially when interaction between features is present.

## AI, ML, DL, Large Language Models, and Generative AI

### Overview

**Figure 4.**
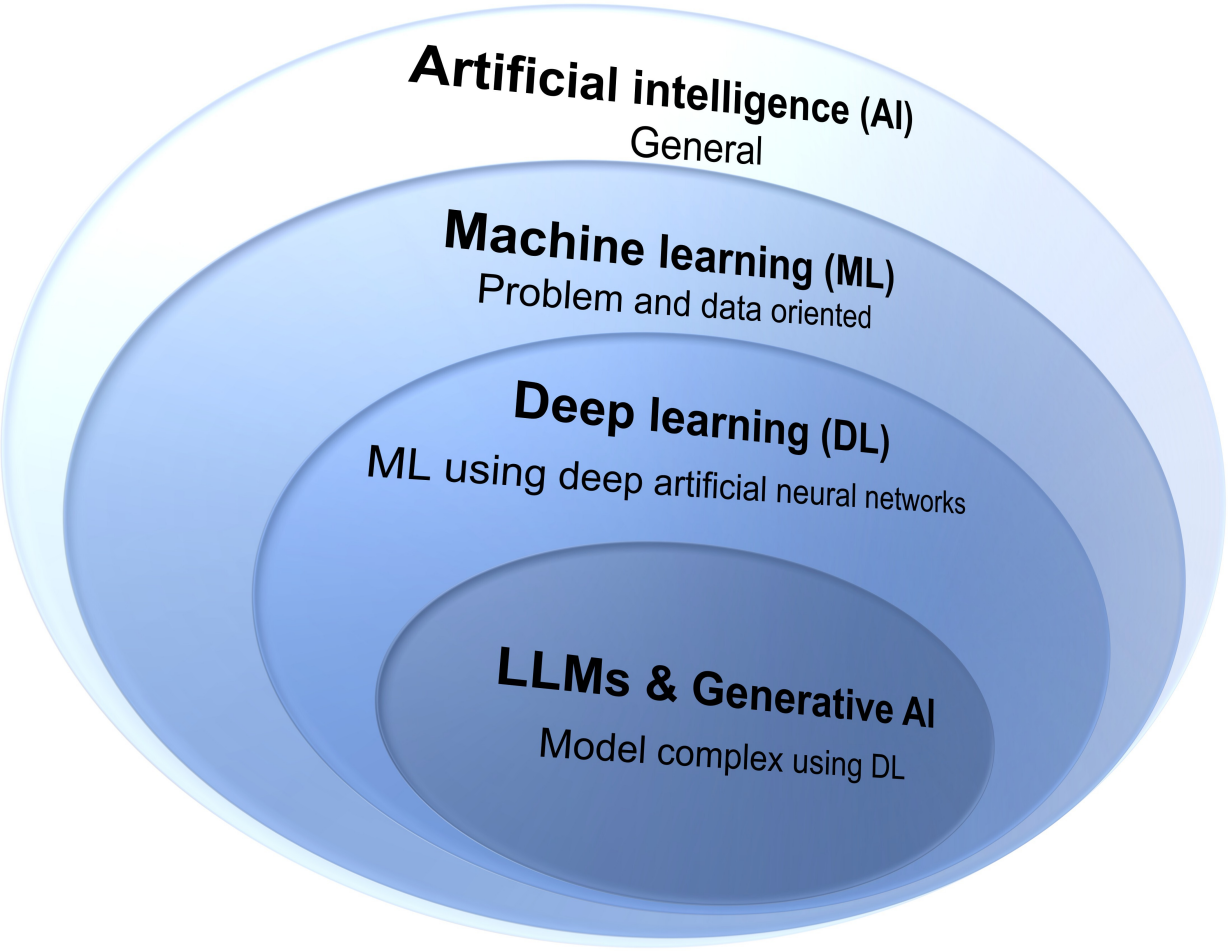
Simplified relationships between artificial intelligence (AI), machine learning (ML), deep learning (DL), large language models (LLMs), and generative artificial intelligence. Modern LLMs and generative AI are still evolving and expanding the capabilities of both DL and ML, potentially surpassing traditional ML.

The terms AI, ML, and DL are often used interchangeably in medicine, psychology, and social science literature, particularly since the emergence of generative AI, and could be potentially confusing for different audiences (Figure 4).

AI encompasses any man-made algorithm or agent (ie, computation) exhibiting aspects of intelligence, such as perceiving, reasoning, and acting [52]. As a field, AI is broad and multifaceted. When referring to an AI agent, most experts expect it to mimic human cognition and behaviors, solve complex problems, and learn from experience [53]. However, AI includes many subfields beyond just learning systems, such as logic systems, symbolic reasoning, or philosophical components.

ML represents a specific subset of AI focusing on algorithms that learn patterns from data to make predictions or decisions. For example, ML has successfully identified or predicted attention deficit, depression, bipolar disorder, suicidality, and substance use disorders and overdose using behavioral, cognitive, and health record data [14-16,22,23,32,54,55]. With the proliferation of big data, ML has become the most visible component of AI, but it does not encompass all aspects of AI.

The defining characteristic of ML is that the model structure and parameters are learned automatically from data, usually high-dimensional, rather than being explicitly programmed. These models can range from relatively simple linear equations and support vector machines to complex deep neural networks.

DL represents a specialized form of ML using multilayer artificial neural networks inspired by the structure and function of the human brain [36]. While this brain-inspired architecture may contribute to confusion between DL and broader AI, DL remains fundamentally a subset of ML focusing on making predictions based on data. The complexity of DL increases when researchers develop advanced architectures such as those used in generative AI applications or federated learning [56], or when applying these models in reinforcement learning scenarios [57]. Nevertheless, these advanced applications still fundamentally operate within the ML paradigm.

ML encompasses various pattern recognition tasks that identify meaningful data structures such as faces, objects, words, or sentiments [37]. This aspect has gained particular prominence with the emergence of large language models (LLMs), which are primarily trained to predict the next term according to a given context. The definition of “large” may change depending on historical considerations, and previous language models could be preprogrammed and might not use DL paradigms [58]. The current versions of LLMs are usually deep neural networks with billions or even trillions of parameters [59]. While generative AI conceptually encompasses any system that creates new content (including rule-based and symbolic approaches), modern generative AI systems predominantly use DL techniques. Historical examples such as ELIZA or procedural content generation in games demonstrate non-ML generative approaches, but today’s state-of-the-art generative AI relies fundamentally on DL architectures. Modern LLMs and generative AI are still evolving and expanding the capabilities of both DL and ML, potentially surpassing traditional ML, especially with the emerging agentic AI and world models [60,61].

The growing popularity of DL has sparked discussions about “explainable AI” [62,63], reflecting concerns about the interpretability of complex models. While many ML algorithms provide reasonable transparency regarding influential variables, DL approaches often function as “black boxes” with limited interpretability. However, interpretability exists on a spectrum—linear ML methods, LASSO regression, and support vector machines typically offer greater insight into the importance of specific variables for prediction than deep neural networks.

It is important to note that coefficients or importance scores from ML models should not be directly interpreted as statistical indicators (eg, odds ratios). Separate hypothesis-driven analyses dedicated to interpretability are usually necessary to complement ML predictions [54,64,65]. These hybrid approaches can be crucial for understanding AI models and their predictions in clinical and social contexts.

### Recommendations for the Use of AI, ML, DL, and LLMs

Although the recent advancement of LLMs and generative AI [66,67] has further blurred boundaries between them and AI, ML, and DL, we recommend using the most specific term possible when describing research applications. Studies using LLMs, generative AI, or other DL variants should be labeled accordingly rather than using the broader term “ML.” Similarly, studies using various ML algorithms to predict clinical outcomes should be described as “ML” rather than the more general term “AI.” Although AI is a buzzword, its general nature can be misleading without specific methodological details.

Due to the generative nature of LLMs and generative AI models that extends beyond traditional prediction tasks, it is important to specify their precise application, such as generating synthetic data (text, images, and videos), simulating human interactions (structured interviews, psychological assessments, diagnostics, cognitive-behavioral and group therapies, patient education, virtual patients, or health service providers), synthesizing knowledge from scientific literature, providing decision support with treatment alternatives, designing research protocols and experiments, improving accessibility through translation and simplification, and assisting with other specialized tasks. This specificity helps maintain terminological clarity and prevents confusion about the model’s purpose, capabilities, and appropriate evaluation metrics.

## Conclusions

In this paper, we have systematically addressed common terminological confusions surrounding AI applications in medicine, psychology, and social sciences. We have provided clarification on key terms and concepts that are frequently misused or misunderstood in the literature, with a particular focus on “prediction”—a term that systematic reviews have shown is often incorrectly applied to association studies. We have established practical recommendations for when and how the term “prediction” should be used, emphasizing the importance of specifying the nature of prediction in a study, especially the use of “prospective prediction” for future prediction. We have also elucidated validation procedures essential for ensuring model generalizability and predictability and provided recommendations for using prospective, internal, and external validation.

Our discussion of overfitting and regularization has highlighted the advantages of ML approaches over traditional regression methods for developing generalizable models. By clarifying the relationships between features, independent variables, predictors, risk factors, and causal factors, we have provided researchers with a recommendation for more precise communication about model inputs and their interpretations.

Finally, we have delineated the hierarchical relationships between AI, ML, DL, LLMs, and generative AI, establishing a clear approach for discussing these increasingly prevalent technologies.

Maintaining terminological precision has become increasingly important and challenging as AI technologies continue to evolve rapidly. We hope that the recommendations provided in this tutorial will facilitate clearer communication among researchers and clinicians across different disciplines and between the research community and the public, advancing the rigorous and effective application of AI and ML in medicine, psychology, and social sciences, leading to improved research quality and, ultimately, better health and social outcomes (Multimedia Appendix 2).

## Supplementary material

10.2196/66100 Multimedia Appendix 1 Prospective internal or external validation.

10.2196/66100 Multimedia Appendix 2 Glossary of terms.

## Abbreviations

AI: artificial intelligence
DL: deep learning
LASSO: Least Absolute Shrinkage and Selection Operator
LLM: large language model
ML: machine learning
