# Supplementary figures and images for: AI and Machine Learning Terminology in Medicine, Psychology, and Social Sciences: Tutorial and Practical Recommendations
Source: J Med Internet Res. 2025 Aug 18;27:e66100. doi: 10.2196/66100 (PMC12360722; doi:10.2196/66100)

Table S1. Prospective internal/external validation.


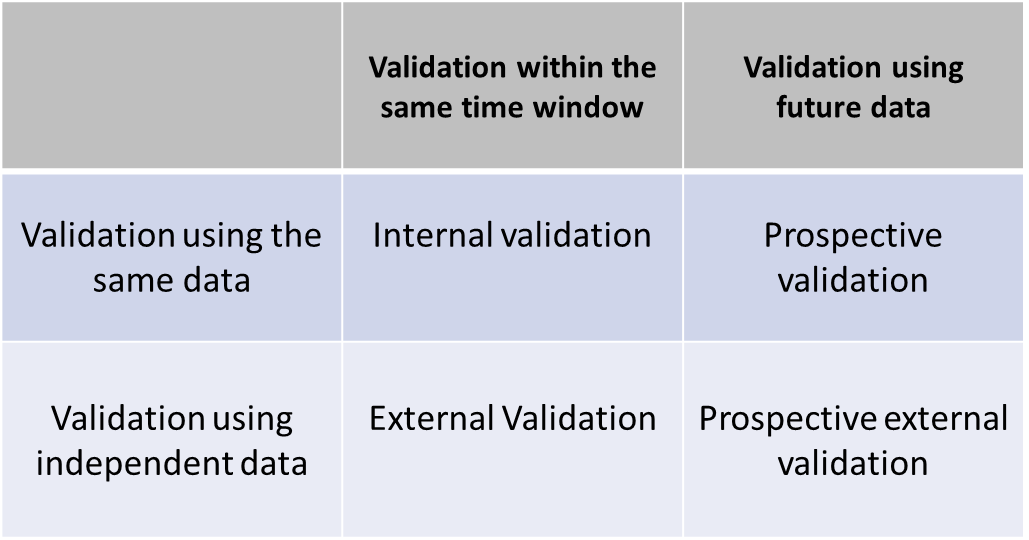

Supplement: Multimedia Appendix 1 [file jmir-v27-e66100-s001.docx]
