# Supplementary material for: AI and Machine Learning Terminology in Medicine, Psychology, and Social Sciences: Tutorial and Practical Recommendations
Source: J Med Internet Res. 2025 Aug 18;27:e66100. doi: 10.2196/66100 (PMC12360722; doi:10.2196/66100)
Supplement: Multimedia Appendix 2 [file jmir-v27-e66100-s002.docx]

**Glossary of Terms**

**Artificial Intelligence (AI):** A broad field encompassing any man-made algorithm or agent that exhibits components of intelligence, including learning, reasoning, and problem-solving capabilities.

**Association:** A statistical relationship where one variable provides information about another, not necessarily implying prediction or causation.

**Causal Factor:** A variable that has been experimentally demonstrated to directly influence an outcome through controlled experimental designs.

**Cross-Validation:** A validation technique where data are randomly split into portions, with each portion serving as validation data in turn while the remaining data are used for training.

**Deep Learning (DL):** A specific form of machine learning using multilayer artificial neural networks inspired by the structure and function of the human brain.

**External Validation:** Validation of a model using a dataset that is independent from the development dataset, typically collected from a separate cohort or another geographical region or facility.

**Explainable AI:** Approaches that aim to make AI systems understandable to humans, revealing how models make decisions and predictions.

**Feature:** An input variable used in machine learning models, referring collectively to all variables in the data, usually excluding the target variable.

**Feature Space:** The set of all possible values of features in a machine learning model.

**Federated Learning:** A machine learning approach where multiple entities collaborate to train a model without sharing their data, instead sharing model updates.

**Generative AI:** AI systems that can generate new content (text, images, etc.) based on patterns learned from training data, often implemented using deep learning techniques.

**Independent Variable:** A variable in statistical models that is (ideally) independent of other variables and used to explain or predict the dependent variable(s).

**Individualized Prediction:** Predictions made at the individual level, as opposed to predictions of outcomes at the group or population level.

**Internal Validation:** Evaluation of a model’s performance using the same data that was used in the development process, often through holding a portion of data out.

**Large Language Models (LLMs):** Advanced deep learning models trained on vast text data that can generate human-like text and perform various language tasks.

**Machine Learning (ML):** A subset of AI focused on algorithms that learn patterns from data to make predictions or decisions without being explicitly programmed.

**Overfitting:** A phenomenon where a model learns the training data too well, capturing both signal and noise, resulting in poor generalization to new data.

**Pattern Recognition:** A specific task in machine learning focused on recognizing meaningful patterns in data, such as faces, words, or sentiments.

**Personalized Medicine:** A medical approach that uses individual patient characteristics to optimize treatments, typically involving multiple individualized prediction models for various treatment options using multimodal data.

**Precision Medicine:** Similar to personalized medicine, an approach that tailors health care decisions to individual patients based on their characteristics, often using prediction models.

**Predictor:** A feature or independent variable confirmed through testing or validation to have predictive value for an outcome.

**Prospective Prediction (Forecasting):** Predictions specifically aimed at future outcomes, emphasizing the temporal aspect of prediction.

**Prospective Validation:** Validation of a model using data collected in the future after model development, often involving longitudinal collection from the same or another independent cohort.

**Regularization:** Techniques used in machine learning to prevent overfitting by constraining model parameters, such as controlling coefficient magnitudes in regression models.

**Reinforcement Learning:** A type of machine learning where agents learn to make decisions by receiving rewards or penalties based on their actions.

**Risk Factor:** A variable associated with increased probability of an outcome, typically identified through association studies rather than predictive modelling.

**Support Vector Machine (SVM):** A supervised learning algorithm that classifies data by finding the hyperplane that best separates different classes in the feature space.

**Testing:** Evaluation of a model’s performance on data not used during training, often used informally but less precise than specific validation terms.

**Validation:** The process of evaluating a model’s performance on data not used during training to estimate how it would perform on new, unseen data.
